# Supplementary material for: Chromothripsis during telomere crisis is independent of NHEJ, and consistent with a replicative origin
Source: Genome Res. 2019 May;29(5):737–49. doi: 10.1101/gr.240705.118 (PMC6499312; doi:10.1101/gr.240705.118)
Supplement: Supplemental Material [file supp_gr.240705.118_Supplemental_file_1.zip › contigs/annotated_contigs/DB53/contig.2.DB53_length_788_mean_cov_4.89847715736.docx]

**DB53_length_788_mean_cov_4.89847715736**

```
CTGAATCATCCCTTTATCATTACATAATGACTTTCTTTGTTCATTTTCATAGTTATTAATGTAAAGTGTATTTTATCTTGCATAAGTAT
 >chr8:131942103-131942512 + E=3e-228 p=5e-02
AGGTATTCCTGCTTTCTTTTGTTTTTTATTTGCAGGTAATACCTTTTCTATCCTTCACTTTCAATCTATGTGTGTCCTACAGGTAAAGT

GAGCCTTTGTAAGCAGCATATAGTTGAGTCTTGTTTTTCATCCATTAAGGCACTCTACATTTTTTGATTCTAGAATTTAATGCATTTAC

ATTACATGTAGAGACTTGTTAATTGTTTTCTAGTTGTTTTGTAGATCTTGTGTTACTTCTTCCTTTCCTTTTGTGTTCCTCTGAGGTTA

AGTGATTTTCACTAGTGTTATATTTGGATTCCTTCTTCTACTTTTTTTTTTTT|GAGAATATAGTCTTACTTAGACTCTAAGGAGCAAG
                                                       >chr8:132413853-132414232 - E=4e-
TCGTACTTTATTTCTTTATTTTTTCAATAATGCATAAAACTTTTGGTACTAGAGTTATTTCCACTAGACTCTATGCTGTTTGAATTTCA
204
GGAATGTTTCTTACTCATGTTTATATTTTCTATACCTAGCATAATACCTCACACATATTATGTCTCAATAAAGAGTTGTTGAACTAATG

AGTGAATTAATTCAAACATGACACCATTAAATAAATGGAAACAGTGATTTATAATAAGATATTGGATCTTGCATCATAGAGAAATATGC

TGTCTCAGGATTCTTTTCCATCACCTTCAATACCAGGCATACACAGGGCTCGTTAACAATGTCAGATGCATCCCTCA
```
